# Changes in Sexual Behavior and Attitudes Across Generations and Gender Among a Population-Based Probability Sample From an Urbanizing Province in Thailand

**DOI:** 10.1007/s10508-014-0429-5

**Published:** 2014-11-18

**Authors:** Teeranee Techasrivichien, Niphon Darawuttimaprakorn, Sureeporn Punpuing, Patou Masika Musumari, Bhekumusa Wellington Lukhele, Christina El-saaidi, S. Pilar Suguimoto, Mitchell D. Feldman, Masako Ono-Kihara, Masahiro Kihara

**Affiliations:** 1 Department of Global Health and Socio-Epidemiology, Kyoto University School of Public Health, Yoshida-Konoe cho, Sakyo Ku, Kyoto, 6068501 Japan; 2 Institute for Population and Social Research, Mahidol University, Nakhon Pathom, Thailand; 3 Department of Medicine, University of California, San Francisco, CA USA

**Keywords:** Sexual attitudes, Sexual behavior, Thailand, Multistage sampling, Sex survey

## Abstract

Thailand has undergone rapid modernization with implications for changes in sexual norms. We investigated sexual behavior and attitudes across generations and gender among a probability sample of the general population of Nonthaburi province located near Bangkok in 2012. A tablet-based survey was performed among 2,138 men and women aged 15–59 years identified through a three-stage, stratified, probability proportional to size, clustered sampling. Descriptive statistical analysis was carried out accounting for the effects of multistage sampling. Relationship of age and gender to sexual behavior and attitudes was analyzed by bivariate analysis followed by multivariate logistic regression analysis to adjust for possible confounding. Patterns of sexual behavior and attitudes varied substantially across generations and gender. We found strong evidence for a decline in the age of sexual initiation, a shift in the type of the first sexual partner, and a greater rate of acceptance of adolescent premarital sex among younger generations. The study highlighted profound changes among young women as evidenced by a higher number of lifetime sexual partners as compared to older women. In contrast to the significant gender gap in older generations, sexual profiles of Thai young women have evolved to resemble those of young men with attitudes gradually converging to similar sexual standards. Our data suggest that higher education, being never-married, and an urban lifestyle may have been associated with these changes. Our study found that Thai sexual norms are changing dramatically. It is vital to continue monitoring such changes, considering the potential impact on the HIV/STIs epidemic and unintended pregnancies.

## Introduction

Globalization, driven by technological advances that have increased the interconnectivity of people and accelerated the spread of ideas, information, and perceptions, has resulted in massive social and cultural changes (World Health Organization, 2013). These trends have had a major impact on sexual norms, particularly among young people in middle-income countries. Widespread industrialization has led to more youth, especially women, seeking higher education and employment which, in turn, has resulted in delayed marriage, increasing cohabitation, and higher rates of premarital sex (Bozon & Kontula, 1998; Weniger & Brown, 1996). At the same time, occupational demands have brought about changes in family structure, such as the diminishing role of the extended family in provision of care and support, and decreasing parental monitoring (Podhisita Chai, Xenos Peter, & Varangrat Anchalee, 2001; Vichit-Vadakan, 1994). Collectively, younger generations have been exposed to rapid changes in social norms, urban values, and intense sexual stimuli through the mass media and the Internet, leading to greater sexual freedoms and evolving norms in sexual behavior and attitudes (Friedman, 1992; Tangmunkongvorakul, Kane, & Wellings, 2005). Thailand, as a newly emerging industrialized country with increasing urbanization and rapid globalization (affirmed by the World Bank’s recent upgrade from a lower-middle to an upper-middle income economy) (The World Bank, 2013) is unlikely to be immune to these changes in sexual norms.

Additionally, distinctive to Thailand is the historical context of Thai sexuality in relation to the human immunodeficiency virus (HIV) epidemic in the 1980s. The widespread publicity of the epidemic and subsequent prevention campaigns, such as the “100 % Condom Campaign” (Rojanapithayakorn & Hanenberg, 1996), is believed to have had an influence on Thai sexual behavior and attitudes, particularly among men. In part out of fear of HIV, Thai men have shifted from patronage of commercial sex workers (CSWs) to their female peers and non-commercial casual partners (Hanenberg & Rojanapithayakorn, 1998; VanLandingham & Trujillo, 2002). Coupled with greater sexual freedom among Thai women in the midst of rapid social and cultural transformations (Morrison, 2004; Vichit-Vadakan, 1994), it is plausible that sexual norms, in terms of both behavior and attitudes, have been changing significantly, and differentially, by gender and generation.

However, empirical evidence of these changing sexual attitudes and practices in Thailand is lacking from population-based surveys. As suggested by Fordham (2005), studies on sexual behavior and attitudes in Thailand were mainly motivated by the threat of the HIV epidemic and were generally limited to understanding and monitoring risky sexual behavior of high risk populations (Mills et al., 1997), such as vocational school students (Allen et al., 2003; van Griensven et al., 2001; Whitehead et al., 2008), male conscripts (Nelson et al., 1996), men who have sex with men (Beyrer et al., 1995; Li et al., 2009), and CSWs and their male clients (Celentano et al., 1993; Jenkins et al., 1999; Morris, Pramualratana, Podhisita, & Wawer, 1995; VanLandingham, Somboon, Sittiitrai, Vaddhanaphuti, & Grandjean, 1993).

The few studies to date that have examined sexual behavior among the Thai general population have significant methodological limitations. The first limitation is the mode used to administer the questionnaire. Most studies have used face-to-face interviews (Chamratrithirong, Kittisuksathit, Podhisita, & Sabaiying, 2007; Sittitrai, Phanuphak, Barry, & Brown, 1992) that afforded less privacy and anonymity and thus likely increased motivational bias (Schroder, Carey, & Vanable, 2003). The second limitation is the type of study setting. Although Lertpiriyasuwat, Plipat, and Jenkins (2003) used self-administered questionniares, their study was conducted in an area with a predominantly rural population, which does not meet our research objective, as we hypothesized that changes in sexual norms are probably most prominent in an urban setting.

To address these shortcomings of prior research, we conducted a population-based cross-sectional probability sample survey among the general population in an urban setting, covering participants from a wide range of birth cohorts as well as employing a self-administered computer-assisted data collection procedure. This study design provided a cross-sectional picture of sexual behavior and attitudes that, at least in part, may reflect the secular trends of changes across genders and generations, especially young people (Johnson, Wadsworth, Wellings, & Field, 1994). Furthermore, taking into consideration public health threats of increasing STIs (Bureau of Epidemiology, 2013), unintended pregnancies (Ministry of Social Development and Human Security, 2010), and the projected re-emergence of the HIV epidemic in Thailand (Commission on AIDS in Asia, 2008), our study also aimed to estimate the pattern and the prevalence of risky sexual behaviors that are major determinants of HIV/STI transmission and other sexual health outcomes, including the diversity of sexual behaviors (Johnson et al., 2001; Turner, Danella, & Rogers, 1995).

## Method

### Participants

The primary aim of this study was to examine and test for differences in the proportion of adolescent sex across generations and gender. Due to limited information on the proportion of adolescent sex among older age cohorts in Thailand, we calculated our sample size using the data of a 1999 nationwide survey in Japan, a country once under conservative sexual norms that later experienced a sharp increase in adolescent sex from the mid-1990s. The findings of the survey portrayed a clear transition of sexual norms in Japan, reflected by increasing rates of adolescent sex across age groups; 18 % in age 18–24, 4–6 % in age 25–44, and 1–2 % in age 45 and above (Ono-Kihara, 2011). As Thailand shares similar Asian conservative sexual values, we speculated that Thailand would be going through a similar transition following rapid urbanization. Available information shows that the sexual experience rate of Thai adolescents is approximately 10–20 % in both genders (Bureau of Epidemiology, 2011; Ruangkanchanasetr, Plitponkarnpim, Hetrakul, & Kongsakon, 2005), comparable to the 1999 nationwide Japanese data (18 % in the 18–24 age group) (Ono-Kihara, 2011). We therefore used this information to assume the proportion of adolescent sex in different cohorts for both genders in Thailand; 20 % in age 15–24, 5 % in age 25–44, and 1 % in age group over 45 years old. To detect the difference between age group (1) 15–24 versus 25–44, (2) 25–44 versus over 45, and (3) over 45 vs. 15–24 with statistical significance (α = 0.05, β = 0.2), the total sample size required for these comparisons were (1) 154, (2) 570, and (3) 80, respectively. Taking into consideration the subgroup analyses, complex study design effects of D = 2.0 (Family Health International, 2000) and a response rate of 80 % as reported by the latest National Sexual Behavior Survey in Thailand in 2006 (Chamratrithirong et al., 2007), our final sample size was estimated at 2,500 to cover men and women age 15–59 years.

Overall, 85.5 % (2,138/2,500) participated in the study. By residential area, it was 80.3 % (1,004/1,250) in urban areas and 90.7 % (1,134/1,250) in rural areas. Since participation was generally refused at the doorstep, we had no further information (age and gender) of all eligible participants of that particular household and hence no information of those who refused.

### Procedure and Measures

#### Study Setting

Given that the changes in sexual behavior and attitudes are most prominent in urbanized areas, we selected Nonthaburi province as our study setting. Second to Bangkok, Nonthaburi is the most urbanized and densely populated province in the central region with an estimated population of 1.14 million (996,686 residents [aged 15–59 years]) at the end of 2012 (Department of Provincial Administration, 2013). The province is divided into 6 districts (Amphoe) which, in turn, are divided into 52 communes (Tambon) and further divided into 328 villages (Mubaan) (Nonthaburi Office of Governor, 2011).

#### Survey Instrument

A self-administered structured questionnaire was created based on a review of the Thai and the international literature. To improve the initial draft, we used a qualitative data collection approach which involved conducting focus group discussions (FGDs) among 20 local participants recruited through purposive sampling. Our FGDs had several aims: to resolve language discrepancies of the translated draft, to test for face validity of the questionnaire items, and to discuss various aspects of sexual behavior and attitudes, investigating new ideas that could contribute to the improvement of the questionnaire. The modified draft was then converted into an electronic format compatible with an Internet-enabled tablet, designed to be user-friendly even for those participants not familiar with electronic devices. Using the tablets, we assessed for test–retest reliability in a 2-week interval in another set of 30 Nonthaburi residents. Kappa coefficients were calculated for dichotomous variables and intraclass correlation coefficients for non-dichotomous variables (Schroder et al., 2003; Streiner & Norman, 2008). All variables demonstrated good reliability of 0.60–1.00 (all *p*s < .05). Lastly, we carried out the final pretest of the tablet-based questionnaire among a separate set of 40 local residents to test for skip logic and final flow of the software. All individuals who participated in the instrument development phase were recruited from locations outside of our designated sampling areas and were not included in the main survey.

#### Study Design and Sampling

**Fig. 1:**
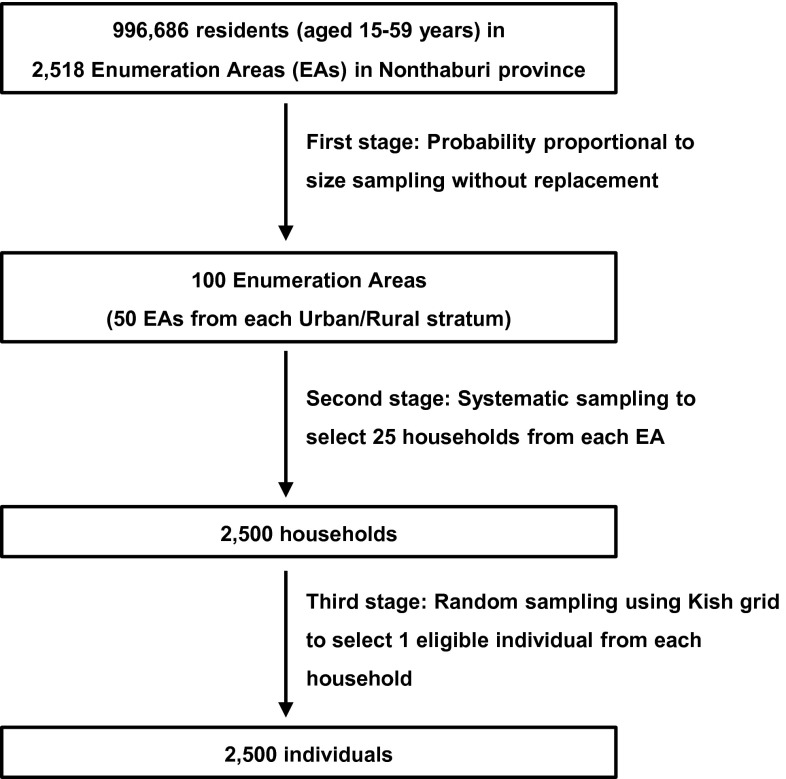
Sampling procedures

The survey was a cross-sectional study which employed a three-stage, stratified, probability proportional to size (PPS), clustered sampling as depicted in Fig. 1. A list of study sampling clusters, or Enumeration Areas (EAs), was provided courtesy of the Thailand National Statistical Office. In the first stage, 100 EAs (50 from each urban and rural stratum) were systematically selected by PPS sampling without replacement (Family Health International, 2000; United Nations, 2005) using the latest sampling frame of the 2010 National Population and Housing Census for Nonthaburi province. In the second stage, within each selected EA, we conducted field work listing to make a record of all eligible households in order to have an accurate sampling frame. Those who had been in their dwelling less than 1 month at the time of the survey and visitors to the province were not eligible for the study. A total of 25 households were then selected by systematic sampling from each EA (Family Health International, 2000). In the final stage, within each selected household, a list of all eligible members was created during the visit to the household. Field staff briefly introduced the survey, explained the research objectives, and sought permission to list all eligible members in the household in order of decreasing age to prepare for participant selection procedure. Taking into consideration potential correlated attitudes within the household, only 1 individual was selected per household (Kish, 1949). We used the Kish grid (Kish, 1949) for selection of the main participant to prevent biases towards people who may be more cooperative or are home more often (Clark & Steel, 2007). The grid included a selection table that gave nearly equal probability of selection to each household member. If the selected individual was unavailable, an appointment was made for the next visit. A non-response was considered after three unsuccessful attempts.

Field work for the data collection was carried out from October to December 2012 by 14 field staff. To ensure high quality data collection, we recruited staff with at least a Bachelor’s degree and with prior field survey experience. All field staff attended a 1-week intensive training to learn about the study research objectives and methods, how to use the research tool, how to formally introduce themselves to potential participants and how to ensure confidentiality in participation to obtain informed consent. Furthermore, to assist those individuals with limited reading proficiency, field staff carried an additional printout of all screenshots of the electronic questionnaire to read out loud and guide participants without seeing their responses. Data collection took place inside participants’ households or nearby areas as participants preferred, where they could complete the questionnaire in privacy. Field staff waited close by in case any assistance was needed. The questionnaire software was programmed to automatically upload the results to the main server in real-time. Field staff had no access to the responses.

The research protocol was approved by the Committee for Research on Human Subjects of Kyoto University, Japan (E1320) and The Committee for Research Ethics (Social Sciences) of Mahidol University, Thailand (2012/072.0103 [B2]). All participants provided verbal informed consent prior to participation. For those participants whose age was less than 18 years, a separate parental consent was also obtained. Participants received a small bag containing HIV/AIDS related educational pamphlets to acknowledge their participation.

### Statistical Analysis

Descriptive statistical analyses were carried out using the Complex Sample module of SPSS version 21 to account for the effects of multistage sampling, stratification, clustering, and weighting. Sample weights were calculated for the adjustment of (1) differential selection probabilities at each sampling stage, (2) non-response in each EA, and (3) post-stratification to the latest 2010 census estimates to correct for differences between our sample and the provincial urban/rural population estimates. The product of all sample weights was further standardized such that the total weighted sample was equal to the total unweighted sample (Macro International Inc., 1996), constituting the final weight used in descriptive statistical analyses. To examine the cohort differences, participants were segregated by age: 15–19, 20–24, 25–34, 35–44, 45–54, and 55–59.

To test for significant differences across age groups within gender (young-old men and young-old women), tests of independence of row and column (Rao–Scott adjustment to chi square) were performed for categorical variables. The standard chi square test inflates the type I error rate when a strong, positive intra-cluster correlation is present (Roberts, Rao, & Kumar, 1987). The Rao–Scott adjustment to the chi square statistic takes the complex sample design into account and, therefore, is a more accurate indicator of the statistical significance of the relationship between the row and the column variables than the regular chi square statistic (Berkeley, 2014; Lee & Forthofer, 2006; Rao & Scott, 1981). Significance is calculated from a variant of the second-order Rao–Scott adjusted chi square statistic, using adjusted *F* and its non-integer degrees of freedom (Rao & Scott, 1984). For continuous variables, one-way analysis of variance (ANOVA) was performed. To test for significant differences between genders within age group (young men–women and old men–women), tests of independence of row and column (Rao–Scott adjustment to chi square) were performed for categorical variables and independent sample *t* test for continuous variables.

Additionally, to evaluate the trend with age and gender by adjusting the possible confounding of other important demographic variables (education, marital status and residential areas), multivariate logistic regression analyses were performed using unweighted data with each sexual behavior or attitude as an outcome variable. In preliminary analyses, results of age group 15–19 were similar to those of 20–24, so we collapsed the age groups into 15–24 to facilitate data interpretation. Similar procedures were carried out for age group 25–34 and 35–44 (collapsed into 25–44), and 45–54 and 55–59 (collapsed into 45–59). Likewise, results for attitudes toward premarital sex of male and female adolescents were quite similar, so we combined these variables to create a new variable “premarital sex of adolescents” to facilitate data interpretation. Similar procedures were performed for the same reason to the attitudes toward premarital sex of middle-aged men and women (combined into “premarital sex in middle-aged”) and attitudes toward buying and selling sex (combined into “sex trade”). For the analysis of “ever had sex,” marital status was excluded from the model due to multicollinearity. “First sex under age 15” was analyzed excluding marital status and education level and was dichotomized into “primary education” and “others” considering the age when sex first occurred. Analysis on “CSW as a first sexual partner” included only sexually experienced men since no women reported having had first sex with CSW. Analysis on the sexual attitude item—“woman carrying condom”—was conducted separately for men and women since the age trend was apparently opposite between the genders.

## Results

### Demographic Characteristics

Mean age of participants was approximately 32 and 34 years for men and women, respectively. As shown in Table 1, there was a clear trend of higher education attainment in younger generations in both genders. In general, men had higher education attainment than women in all age groups except for the age group of 15–34 years where more women than men attended university. The majority of participants of both genders were employees (public and private sectors) and business owners with a lower proportion of farmer/labor, reflecting the characteristics of the study setting which was largely urbanized. Proportions of ever-married increased as age increases and were similar between genders, except for the age group 15–24 where more women were ever married than men (24.9 vs. 8.4 %).

**Table 1:** Sociodemographic characteristics of participants by age group and gender

Characteristics	Age group and gender															
	15–19		20–24		25–34		35–44		45–54		55–59		Total		*F*(df1, df2)	
	M	W	M	W	M	W	M	W	M	W	M	W	M	W	M	W
N (weighted, unweighted)	208, 197	141, 122	175, 151	192, 158	220, 239	262, 249	152, 179	266, 300	166, 181	225, 230	72, 63	60, 69	993, 1010	1145, 1128		
Education															10.94*** (7.96, 772.00)	19.50*** (8.52, 826.02)
≤Primary	10.1	2.2	4.2	8.6	10.4	7.3	13.4	23.9	27.4	40.3	38.8	46.2	14.6	19.3		
Secondary	83.9	92.3	60.6	51.5	52.6	43.2	44.3	43.3	47.3	36.3	35.5	29.0	57.2	48.5		
University	6.0	5.4	35.3	39.9	37.0	49.5	42.2	32.8	25.3	23.4	25.6	24.7	28.2	32.2		
*F*(df1, df2)	3.89^†^ (1.98, 169.92)		1.61 (1.95, 179.49)		2.72 (1.64, 156.14)		3.44^†^ (1.95, 177.78)		2.89 (1.77, 164.73)		0.29 (1.99, 127.42)		6.19^††^ (1.87, 181.30)			
Occupation															16.11*** (17.84, 1730.54)	20.07*** (18.26, 1770.83)
Unemployed^a^	10.2	17.3	9.6	30.3	3.6	28.4	4.3	25.7	4.5	35.8	18.2	53.7	7.3	29.5		
Employee^b^	2.4	2.1	22.4	21.0	48.9	38.5	35.5	27.3	38.0	19.2	24.0	18.5	28.8	23.7		
Business owner	2.4	2.2	13.7	2.5	23.7	21.8	29.5	28.8	29.2	27.6	19.7	18.3	19.0	18.7		
Farmer/Labor	9.8	5.3	12.5	9.0	17.3	9.3	27.4	15.2	19.6	13.4	33.9	7.0	18.0	10.8		
Student	73.3	72.5	39.2	36.3	3.1	0.0	0.0	0.0	0.0	0.0	0.0	0.0	23.0	15.0		
Others	1.9	0.6	0.8	1.0	3.5	2.0	3.2	3.1	8.8	4.0	4.2	2.5	3.9	2.3		
*F*(df1, df2)	1.18 (4.53, 389.60)		6.18^†††^ (4.07, 374.01)		8.02^†††^ (4.54, 431.25)		7.28^†††^ (3.84, 349.59)		11.49^†††^ (3.75, 348.56)		4.97^††^ (3.45, 220.77)		26.94^†††^ (4.64, 449.71)			
Marital status															79.38*** (4.66, 451.90)	53.05*** (4.56, 442.09)
Never married	97.2	86.0	84.9	67.1	56.2	40.2	24.0	15.0	7.0	12.7	3.9	13.6	53.0	37.7		
Ever married	2.8	14.0	15.1	32.9	43.8	59.8	76.0	85.0	93.0	87.3	96.1	86.4	47.0	62.3		
*F*(df1, df2)	13.13^†††^ (1, 86)		11.38^††^ (1, 92)		9.42^††^ (1,95)		4.64^†^ (1, 91)		2.64 (1, 93)		2.9 (1, 64)		39.76^†††^ (1,97)			

All percentages are of column weighted N. Totals of percentages may differ from 100 due to rounding

*M* Men, *W* Women

Significance is based on the adjusted *F* (a variant of the second-order Rao-Scott adjusted chi square statistic) and its degrees of freedom. Significance levels of tests across age groups within gender are represented by *p < .05, **p < .01 and ***p < .001. Significance levels of tests between genders within age group are represented by ^†^ *p* < .05, ^††^ *p* < .01, and ^†††^ *p* < .001

^a^ “Unemployed” includes housewife

^b^“Employee” includes all employees of private and governmental sector

### Sexual Behavior

#### Sexual Experience and Sexual Debut

As shown in Table 2, the reported rate of “ever had sex” increased with age with a steep increase from around 40 % among adolescents to around 70 % among those in their early 20 s. Among the younger age groups (below age 45), men reported higher rates than women. Regarding first sex before the age of 15, there was a clear trend of increasing proportion with decreasing age; from 0 % in the 55–59 age group to 11.4 % in the 15–19 age group in men and from 0.8 to 11.4 % in women. There was no significant gender difference in sex before age 15 in the youngest age group. The mean age at first sex was lower with decreasing age in both genders, along with a decrease in gender difference from 3.7 years in the oldest age group to only 0.3 years in the youngest age group.

**Table 2:** Sexual behavior of participants by age group and gender

Variables	Age group and gender															
	15–19		20–24		25–34		35–44		45–54		55–59		Total		*F*(df1, df2)	
	M	W	M	W	M	W	M	W	M	W	M	W	M	W	M	W
N (weighted, unweighted)^a^	208, 197	141, 122	175, 151	192, 158	220, 239	262, 249	152, 179	266, 300	166, 181	225, 230	72, 63	60, 69	993, 1010	1145, 1128		
Ever had sex (%)^b^	43.7	36.1	87.7	65.4	94.8	84.4	96.9	91.5	99.9	90.0	100.0	88.1	84.4	78.2	55.80*** (3.65, 354.16)	30.66*** (4.29, 416.38)
*F*(df1, df2)	1.00 (1, 86)		17.62^†††^ (1, 92)		9.49^††^ (1, 95)		3.17 (1, 91)		82.17^†††^ (1, 93)		11.56^††^ (1, 64)		8.58^††^ (1, 97)			
First sex before age 15 (%)^b^	11.4	11.4	16.4	2.2	8.9	1.2	4.9	1.1	3.5	1.5	0.0	0.8	8.6	2.6	4.07** (4.63, 448.60)	6.07*** (3.62, 352.46)
*F*(df1, df2)	1.15 (1.94, 166.71)		13.39^†††^ (1.98, 182.06)		12.64^†††^ (1.97, 187.21)		3.82^†^ (1.98, 179.90)		3.30^†^ (1.66, 154.31)		13.58^†††^ (1.94, 124.34)		13.58^†††^ (1.90, 183.82)			
N (weighted, unweighted)^c^	89, 86	45, 38	150, 129	123, 103	206, 225	211, 208	143, 170	231, 267	161, 174	196, 195	71, 62	51, 58	820, 846	857, 869		
Mean age at first sex (SE)	15.3 (0.2)	15.0 (0.2)	16.4 (0.3)	18.1 (0.2)	18.3 (0.3)	21.0 (0.3)	19.0 (0.3)	22.3 (0.3)	19.7 (0.4)	23.1 (0.5)	20.3 (0.5)	24.0 (0.8)	18.2 (0.2)	21.3 (0.2)	40.05*** (5, 93)	127.21*** (5, 93)
*t*(df)	1.00 (57)		−5.54^†††^ (85)		−6.48^†††^ (93)		−7.34^†††^ (91)		−5.71^†††^ (92)		−4.40^†††^ (62)		−12.59^†††^ (97)			
First sex before age 15 (%)^b^	26.9	37.6	19.1	3.5	9.4	1.4	5.3	1.3	3.6	1.7	0.0	0.9	10.4	3.5	8.00*** (4.61, 446.92)	19.70*** (3.61, 349.67)
*F*(df1, df2)	0.48 (1, 58)		10.88^††^ (1, 86)		11.98^††^ (1, 93)		4.20^†^ (1, 91)		0.46 (1, 92)		1.36 (1, 62)		14.42^†††^ (1, 97)			
Type of first sexual partner (%)^d^															11.97*** (10.97, 1063.66)	15.18*** (7.51, 727.97)
Spouse	1.4	11.5	0.3	38.7	4.8	46.3	10.8	73.7	16.5	87.6	24.1	93.3	8.6	63.0		
Bf/Gf	89.6	88.5	80.8	59.0	74.0	48.9	53.6	24.6	31.1	9.2	29.9	4.9	61.1	34.2		
Casual	9.0	0.0	19.0	2.4	18.0	4.8	22.0	1.7	18.8	3.2	13.8	1.7	17.7	2.8		
CSW	0.0	0.0	0.0	0.0	3.2	0.0	13.6	0.0	33.5	0.0	32.2	0.0	12.5	0.0		
*F*(df1, df2)	4.44^†^ (1.99, 115.95)		48.26^†††^ (1.64, 141.07)		18.97^†††^ (2.45, 227.52)		46.75^†††^ (2.81, 255.34)		46.34^†††^ (2.84, 260.89)		19.25^†††^ (2.90. 179.81)		143.12^†††^ (2.79, 270.24)			
Mean number of lifetime sexual partners (SE)	5.4 (0.8)	5.0 (2.1)	10.6 (1.5)	3.6 (0.8)	9.0 (0.9)	2.8 (0.9)	12.3 (1.7)	1.7 (0.3)	18.4 (5.7)	1.3 (0.1)	15.0 (5.6)	1.3 (0.1)	11.9 (1.3)	2.3 (0.3)	4.98*** (5, 93)	3.19* (5, 93)
*t*(df)	0.20 (58)		4.16^†††^ (85)		4.50^†††^ (93)		5.96^†††^ (91)		3.01^††^ (92)		2.46^†^ (62)		7.11^†††^ (97)			
Number of lifetime partners (%)^d^															1.60 (14.06, 1363.33)	4.16*** (13.59, 1318.59)
1	17.1	45.8	16.8	51.2	12.0	55.9	13.6	68.3	17.4	83.7	32.3	79.3	16.6	65.8		
2	16.2	19.7	8.5	18.8	10.2	22.3	11.9	22.1	11.1	10.9	9.4	16.3	10.9	18.7		
3–4	29.2	18.9	19.5	16.0	20.7	16.7	24.4	8.4	17.8	4.2	17.0	4.3	21.1	10.9		
5–9	26.2	8.7	19.6	9.2	30.3	3.5	24.0	0.6	19.5	1.0	13.7	0.0	23.2	3.0		
≥10	11.3	6.8	35.7	4.9	26.8	1.7	26.2	0.5	34.3	0.2	27.6	0.0	28.2	1.6		
*F*(df1, df2)	2.82^†^ (3.22, 186.80)		11.57^†††^ (3.92, 337.39)		27.55^†††^ (3.43, 318.99)		44.31^†††^ (3.29, 299.69)		45.07^†††^ (3.32, 305.44)		7.12^†††^ (3.84, 237.87)		115.15^†††^ (3.48, 337.16)			
In the past 12 months^b^																
Bought sex (%) ^b^	1.8	0.0	5.0	1.4	12.5	0.2	12.3	0.5	7.4	0.0	2.4	0.0	8.1	0.4	3.25** (4.59, 444.87)	0.77 (3.64, 353.26)
*F*(df1, df2)	1.07 (1, 58)		1.51 (1, 86)		79.15^†††^ (1, 93)		43.45^†††^ (1, 91)		13.04^†††^ (1, 92)		1.26 (1, 62)		59.98^†††^ (1, 97)			
Sold sex (%)^b^	0.0	1.9	0.7	1.4	0.3	0.0	0.5	0.4	0.3	0.0	0.0	0.0	0.3	0.4	0.31 (3.35, 325.37)	1.36 (4.14, 401.46)
*F*(df1, df2)	1.95 (1, 58)		0.27 (1, 86)		1.02 (1, 93)		0.03 (1, 91)		1.21 (1, 92)		NA		0.06 (1, 97)			
Had casual sex (%)^b^	31.6	9.0	29.7	3.2	21.3	2.5	9.3	0.9	7.2	0.0	2.1	0.0	17.4	1.8	7.82*** (4.21, 408.34)	3.66* (4.55, 441.66)
*F*(df1, df2)	7.74^††^ (1, 58)		23.78^†††^ (1, 86)		25.53^†††^ (1, 93)		34.24^†††^ (1, 91)		8.48^††^ (1, 92)		0.83 (1, 62)		132.19^†††^ (1, 97)			
Had sex with regular partner (%)^b^	60.0	80.1	76.6	81.8	82.7	85.0	81.9	82.7	72.3	62.2	72.1	39.7	76.0	75.8	3.46* (4.68, 454.26)	10.50*** (4.56, 442.27)
*F*(df1, df2)	4.16† (1, 58)		1.00 (1, 86)		0.24 (1, 93)		0.04 (1, 91)		2.42 (1, 92)		11.57^††^ (1, 62)		0.01 (1, 97)			

*M* men, *W* women, *SE* standard error, *Bf* boyfriend, *Gf* girlfriend. CSW commercial sex worker

Significance levels for tests across age groups within gender are represented by *p < .05, **p < .01, and ***p < .001. For categorical variables, significance is based on the adjusted *F* (a variant of the second-order Rao–Scott adjusted chi square statistic) and its degrees of freedom. The adjusted *F* is a variant of the second-order Rao-Scott adjusted chi square statistic. For continuous variable, significance is based on the one-way analysis of variance (ANOVA). Significance levels for tests between genders within age group are represented by ^†^ *p* < .05, ^††^ *p* < .01, and ^†††^ *p* < .001. For categorical variables, significance is based on the adjusted *F* (a variant of the second-order Rao-Scott adjusted chi square statistic) and its degrees of freedom. The adjusted *F* is a variant of the second-order Rao-Scott adjusted chi square statistic. For continuous variables, significance is based on independent sample *t*-test

^a^All participants

^b^Percentages are of those who responded “Yes” to the question only

^c^Only sexually experienced participants. Sample size varies slightly across variables due to item non-response

^d^Percentages are of column weighted N. Totals of percentages may differ from 100 due to rounding

#### Type of First Sexual Partner

Profiles of the type of first sexual partner were somewhat different between genders in the oldest age groups. The majority of women reported that their spouse was their first sexual partner while the majority of men reported having had first sex with non-spouse partners. For men, sex with a CSW accounted for more than 30 % of reported first sex. However, this gender gap diminished prominently with decreasing age as boyfriends or girlfriends were being reported among the majority in both genders; close to 90 % of both male and female adolescents in the youngest age group.

#### Lifetime Sexual Partners

Mean cumulative number of lifetime sexual partners was higher in men (more than 10) compared with women (less than 2) in the age groups of 35 years and above. Such gender differences diminished with decreasing age, particularly in the youngest age group where both genders reported approximately a similar mean of 5 partners. Trend of multiple sexual partnership (more than one lifetime partner) was clear among women where the proportion increased from 20.7 % (age group 55–59) to 54.2 % (age group 15–19) while the corresponding change was only from 67.7 to 82.9 % of men of respective age groups.

#### Type of Sexual Partner in the Past 12 months

The majority of the sexual partners in the past 12 months consisted of regular partners of both genders. Significant proportions of participants in the older age groups were sexually inactive. More than 12 % of men age 25–44 years reported buying sex in the past 12 months. The proportion of participants selling sex in the past 12 months was 0.3 % in men and 1.6 % in women aged 15–24. Although there was a large gender difference in the proportion of those who had casual sex in the past 12 months, it increased with decreasing age reaching 31.6 and 9.0 % in men and women, respectively.

### Sexual Attitudes

#### Premarital Sex

For items, on sexual attitudes in Table 3, the response categories were “Acceptable,” “Neutral,” and “Unacceptable.” The proportions presented in the table represent only those who responded “Acceptable” to each item. Around 40–50 % of men or women felt that premarital sex in adolescent is acceptable without large differences across age groups, except for women of the older age groups 45–59. There was a consistent tendency of higher proportion of participants accepting premarital sex of male adolescent than that of female adolescent across all age and gender groups. Similarly, in premarital sex of middle-aged men and women, more participants felt that premarital sex in middle-aged men was acceptable than that of women.

**Table 3:** Percentage of participants who responded “Acceptable” to each sexual attitude item by age group and gender

Attitudes	Age group and gender															
	15–19		20–24		25–34		35–44		45–54		55–59		Total		*F*(df1, df2)	
	M	W	M	W	M	W	M	W	M	W	M	W	M	W	M	W
N (weighted, unweighted)	208, 197	141, 122	175, 151	192, 158	220, 239	262, 249	152, 179	266, 300	166, 181	225, 230	72, 63	60, 69	993, 1010	1145, 1128		
Premarital sex (%)																
Male adolescent	48.8	44.2	63.9	54.2	66.3	53.8	48.4	43.4	50.8	37.4	50.8	28.0	55.8	45.7	3.06* (4.48, 434.08)	3.44** (4.50, 436.50)
*F*(df1, df2)	0.44 (1, 86)		1.41 (1, 92)		5.65^†^ (1, 95)		0.66 (1, 91)		3.99^†^ (1, 93)		5.10^†^ (1, 64)		15.73^†††^ (1, 97)			
Female adolescent	39.4	37.9	59.4	50.5	58.6	47.8	40.7	34.5	43.3	31.2	45.3	15.4	48.5	39.0	4.19** (4.49, 435.58)	5.09*** (4.51, 437.68)
*F*(df1, df2)	0.05 (1, 86)		1.09 (1, 92)		4.18^†^ (1, 95)		1.15 (1, 91)		4.32^†^ (1, 93)		12.93^††^ (1, 64)		12.92^††^ (1, 97)			
Middle-aged man	45.5	45.7	66.6	54.9	75.1	64.9	71.3	65.0	77.6	70.6	78.1	62.8	67.4	61.9	10.12*** (4.77, 462.78)	4.47** (4.23, 412.99)
*F*(df1, df2)	0.00 (1, 86)		2.84 (1, 92)		3.85 (1, 95)		1.51 (1, 91)		1.87 (1, 93)		3.08 (1, 64)		4.98^†^ (1, 97)			
Middle-aged woman	45.9	45.0	65.3	53.4	69.9	63.3	68.3	62.6	74.1	66.3	73.8	52.6	64.8	59.3	6.89*** (4.72, 457.839)	3.52** (4.55, 441.09)
*F*(df1, df2)	0.02 (1, 86)		2.80 (1, 92)		1.52 (1, 95)		1.12 (1, 91)		1.99 (1, 93)		5.35^†^ (1, 64)		5.04^†^ (1, 97)			
Sex trade (%)																
Buy sex	18.3	12.9	44.3	20.1	47.7	24.3	43.2	29.9	49.6	25.1	59.5	26.2	41.4	23.8	9.50*** (4.34, 421.31)	2.63* (4.51, 437.50)
*F*(df1, df2)	1.51 (1, 86)		13.12^†††^ (1, 92)		18.70^†††^ (1, 95)		5.66^†^ (1, 91)		11.28^††^ (1, 93)		13.75^†††^ (1, 64)		38.67^†††^ (1, 97)			
Sell sex	15.4	14.0	44.6	16.3	41.9	21.3	41.4	25.8	46.7	24.0	59.1	25.0	38.8	21.3	8.99*** (4.61, 446.99)	1.82 (4.59, 445.61)
*F*(df1, df2)	0.09 (1, 86)		17.58^†††^ (1, 92)		12.46^††^ (1, 95)		8.43^††^ (1, 91)		9.99^††^ (1, 93)		15.17^†††^ (1, 64)		38.93^†††^ (1, 97)			
Homosexual partnership	11.9	28.2	27.6	28.5	25.8	34.6	25.9	28.3	27.1	34.2	16.0	32.0	22.7	31.1	3.04* (4.77, 462.92)	0.75 (4.40, 426.55)
*F*(df1, df2)	10.32^††^ (1, 86)		0.03 (1, 92)		3.54 (1, 95)		0.22 (1, 91)		1.46 (1, 93)		3.24 (1, 64)		15.09^†††^ (1, 97)			
Multiple sexual partnership	16.2	14.6	37.6	13.2	32.2	20.3	27.5	14.7	27.8	17.5	28.3	13.4	28.1	16.2	3.59** (4.77, 462.92)	0.95 (4.76, 461.76)
*F*(df1, df2)	0.13 (1, 86)		21.82^†††^ (1, 92)		5.28^†^ (1, 95)		7.23^††^ (1, 91)		3.29 (1, 93)		3.59 (1, 64)		27.79^†††^ (1, 97)			
Woman carrying condom	40.8	53.9	51.9	49.0	51.9	50.2	54.1	48.7	52.9	44.6	60.1	31.8	50.7	48.0	1.98 (4.82, 467.16)	1.36 (4.45, 431.99)
*F*(df1, df2)	3.60 (1, 86)		0.16 (1, 92)		0.09 (1, 95)		0.84 (1, 91)		2.06 (1, 93)		11.26^†††^ (1, 64)		0.98 (1, 97)			

For all items, the response categories were “Others” (“Unacceptable” and “Neutral”) and “Acceptable”. All data presented in the table are percentages of those who responded “Acceptable” only

*M* men,* W* women

Significance is based on the adjusted *F* (a variant of the second-order Rao–Scott adjusted chi square statistic) and its degrees of freedom. Significance levels of tests across age groups within gender are represented by *p < .05, **p < .01 and ***p < .001. Significance levels of tests between genders within age group are represented by ^†^ *p* < .05, ^††^ *p* < .01, and ^†††^ *p* < .001

#### Sex Trade

Participants expressed similar attitudes regard both buying and selling of sex across all age and gender groups. In age groups above 20 years old, more than 40 % of men and 15 % of women felt that the sex trade was acceptable. However, in those 15–19 years of age, less than 20 % approved of selling sex without large difference between genders.

#### Homosexuality

Approximately 30 % of women and more than 25 % of men felt that homosexual partnership was acceptable except for men of the youngest (11.9 %) and the oldest (16.0 %) age groups.

#### Multiple Sexual Partnership

In general, a higher proportion of men (approximately 30 %) felt that multiple sexual partnership was acceptable than women (approximately 16 %) except men in the youngest age group where only 16.2 % found it acceptable.

#### Woman Carrying Condom

Although roughly 50 % of men and women viewed “woman carrying condom” acceptable, the trend was opposite between the two genders; as age increased, the proportion increased in men but decreased in women.

### Multivariate Analysis

In Table 4, we present results of the multivariate logistic regression of sociodemographic characteristics in association with sexual behavior and attitudes. Results of the multivariate analyses were consistent with the results of the bivariate analyses in terms of the trends with regards to age and gender after adjusting for education, marital status, and residential area. Younger age was significantly associated with higher likelihood of having “first sex <15,” “lifetime multiple sexual partnership,” “first sex with boy/girlfriend,” “had causal sex within the past 12 months,” “had sex with regular partners within the past 12 months,” and “acceptance of premarital sex in adolescents.” In regard to gender, being male was associated with greater odds of all other sexual behaviors and attitudes, with the exception of “first sex with spouse” and “acceptance of homosexual partnership” which were exclusively associated with being female. In addition, multivariate analysis results demonstrated that being never-married and/or higher education were associated with “lifetime multiple sexual partnership,” “boy/girlfriend as first sexual partner,” “bought sex in the last 12 months,” “had sex with casual partner in the past 12 months,” “acceptance of premarital sex of adolescents,” “acceptance of sex trade,” “acceptance of homosexuality,” and “acceptance of multiple sexual partnership,” “women carrying condom,” with the last 5 attitudes showing a dose-dependent association with education level. Residential area was related to sexual behavior and attitudes only for “lifetime multiple sexual partnership” and “boy/girlfriend as first sexual partner” in urban dwellers and “spouse as first sexual partner” among rural residents.

**Table 4:** Multivariate logistic regression of sociodemographic characteristics in association to sexual behavior and attitudes

Outcome variables	Age		Gender	Education		Marital status	Residential area
	15–24 (ref: 45–59)	25–44 (ref: 45–59)	Male (ref: female)	Secondary (ref: ≤primary)	≥University (ref: ≤primary)	Never (ref: ever)	Urban (ref: rural)
	Adjusted odds ratio [95 % confidence interval] *p* value						
Sexual behavior							
Ever had sex^a^	0.09 [0.06, 0.14] <.001	0.96 [0.63, 1.47] 0.859	2.13 [1.65, 2.76] <.001	0.60 [0.38, 0.96] 0.033	0.44 [0.27, 0.72] 0.001	–	1.02 [0.79, 1.31] 0.881
First sex <15^a^	7.83 [3.28, 18.69] <.001	3.94 [1.64, 9.47] 0.002	3.63 [2.25, 5.86] <.001	0.91^b^ [0.47, 1.77] 0.785		–	1.17 [0.77, 1.76] 0.463
Lifetime MSP^c^	1.66 [1.11, 2.47] 0.013	2.16 [1.64, 2.85] <.001	9.17 [7.17, 11.73] <.001	1.46 [1.07, 2.00] 0.016	0.84 [0.60, 1.18] 0.313	1.68 [1.23, 1.87] 0.001	1.48 [1.17, 1.87] 0.001
First sex: spouse^c^	0.20 [0.12, 0.33] <.001	0.41 [0.30, 0.57] <.001	0.05 [0.03, 0.06] <.001	0.54 [0.38, 0.78] 0.001	0.68 [0.46, 1.02] 0.060	0.07 [0.04, 0.12] <.001	0.66 [0.50, 0.87] 0.004
First sex: Bf/Gf^c^	5.85 [3.94, 8.68] <.001	2.93 [2.21, 3.88] <.001	2.25 [1.79, 2.84] <.001	1.68 [1.22, 2.33] 0.002	1.64 [1.16, 2.34] 0.005	4.05 [3.05, 5.38] <.001	1.36 [1.08, 1.70] 0.009
First sex: casual^c^	0.88 [0.50, 1.56] 0.661	1.23 [0.83, 1.84] 0.303	9.18 [5.78, 14.54] <.001	1.22 [0.76, 1.95] 0.415	0.83 [0.49, 1.40] 0.484	0.82 [0.54, 1.23] 0.330	1.00 [0.73, 1.39] 0.979
First sex: CSW^d^	0.15^e^ [0.09, 0.25] <.001		–	0.73 [0.41, 1.30] 0.284	1.16 [0.63, 2.13] 0.634	0.62 [0.34, 1.13] 0.118	1.03 [0.67, 1.60] 0.893
Bought sex past 12 months^c^	0.32 [0.13, 0.77] 0.011	1.13 [0.61, 2.09] 0.694	18.45 [6.66, 51.15] <.001	2.65 [1.01, 6.93] 0.048	2.41 [0.89, 6.49] 0.083	2.31 [1.33, 3.99] 0.003	1.26 [0.79, 2.01] 0.336
Had casual sex past 12 months^c^	1.95 [0.98, 3.89] 0.058	2.02 [1.10, 3.72] 0.023	6.47 [3.86, 10.85] <.001	2.25 [1.08, 4.68] 0.030	1.67 [0.78, 3.60] 0.188	3.36 [2.15, 5.25] <.001	1.40 [0.97, 2.01] 0.070
Had sex with regular partner past 12 months^c^	2.36 [1.62, 3.45] <.001	3.23 [2.44, 4.29] <.001	1.36 [1.07, 1.72] 0.013	1.17 [0.86, 1.60] 0.319	1.35 [0.96, 1.91] 0.086	0.36 [0.26, 0.49] <.001	1.13 [0.90, 1.42] 0.308
Sexual attitudes							
Premarital sex in adolescent	1.16 [0.87, 1.55] 0.323	1.30 [1.04, 1.63] 0.020	1.33 [1.11, 1.58] 0.002	1.33 [1.03, 1.72] 0.028	1.93 [1.46, 2.54] <.001	1.45 [1.16, 1.80] 0.001	0.87 [0.73, 1.04] 0.123
Premarital sex in middle-aged	0.47 [0.35, 0.64] <.001	0.85 [0.66, 1.08] 0.186	1.27 [1.06, 1.54] 0.012	0.80 [0.61, 1.06] 0.115	1.14 [0.84, 1.54] 0.397	1.10 [0.86, 1.39] 0.453	1.00 [0.83, 1.20] 0.959
Sex trade	0.42 [0.31, 0.58] <.001	0.82 [0.65, 1.03] 0.088	2.42 [2.00, 2.92] <.001	1.34 [1.01, 1.77] 0.041	1.72 [1.28, 2.31] <.001	1.26 [0.99, 1.59] 0.057	1.00 [0.83, 1.20] 0.964
Homosexual partnership	0.57 [0.41, 0.79] 0.001	0.87 [0.68, 1.11] 0.275	0.56 [0.46, 0.68] <.001	1.18 [0.89, 1.58] 0.256	1.54 [1.14, 2.10] 0.006	1.49 [0.17, 1.90] 0.001	0.90 [0.74, 1.09] 0.266
Multiple sexual partnership	0.69 [0.49, 0.98] 0.039	0.91 [0.70, 1.19] 0.491	1.88 [1.52, 2.32] <.001	1.21 [0.88, 1.68] 0.244	1.64 [1.16, 2.30] 0.005	1.39 [1.07, 1.81] 0.013	0.94 [0.76, 1.16] 0.584
Woman carrying condom	0.86 [0.64, 1.14] 0.294	1.09 [0.87, 1.36] 0.449	1.03 [0.87, 1.23] 0.714	1.33 [1.04, 1.72] 0.025	1.78 [1.35, 2.34] <.001	1.02 [0.82, 1.27] 0.861	1.04 [0.88, 1.24] 0.652
Men only^f^	0.62 [0.40, 0.96] 0.031	0.87 [0.62, 1.23] 0.439	–	1.21 [0.82, 1.79] 0.340	1.91 [1.25, 2.92] 0.003	1.11 [0.80, 1.54] 0.547	1.10 [0.86, 1.42] 0.458
Women only^g^	1.11 [0.76, 1.64] 0.582	1.29 [0.96, 1.73] 0.091	–	1.41 [1.01, 1.96] 0.043	1.59 [1.11, 2.29] 0.012	1.04 [0.77, 1.40] 0.806	1.00 [0.78, 1.27] 0.996

Analysis was not carried out under complex sample module and does not include weight

*Ref* reference category, *MSP* multiple sexual partner, *Bf* boyfriend, *Gf* girlfriend, *CSW* commercial sex worker

^a^All participants (n = 2,138)

^b^Two categories (“≤primary education” and “Others”)

^c^Sexually experienced participants only (n = 1,715). Sample size varies slightly across variables due to item non-response

^d^Sexually experienced men only (n = 846)

^e^Two categories (“15–44” and “45–59”)

^f^All men (n = 1,010)

^g^All women (n = 1,128)

## Discussion

We report on the first comprehensive, cross-sectional study of age- and gender-segregated differential patterns of sexual behavior and attitudes among the general population of one rapidly urbanizing province in Thailand. We found that young Thai men and women were initiating sex at a substantially younger age and with a higher number of sexual partners as compared to older generations. The cohort differences in the type of first sexual partner also support the notion of a changing context of Thai sexual norms—a shift from CSWs to girlfriends in men and from spouses to boyfriends in women.

The changes were especially profound in young women as reflected by the prominent difference in the proportion of participants who had sexual onset before the age of 15 including a higher cumulative number of lifetime sexual partners. This is in contrast to the reported one sexual partner in women in their 40s and 50s. We also found significant changes in sexual attitudes as more young women approved of premarital sex in adolescents and of women carrying condoms in their bags. Altogether, the gender gap in sexual norms in Thai society seems to be diminishing among younger generations.

Multivariate analyses, adjusted by education level, marital status, and residential area, confirmed these findings and further demonstrated that these sexual behaviors and attitudes were associated with higher education, being never-married, and, in part, with urban residence, suggesting that urbanization and contemporary social change may be contributing to the change in Thai sexual norm.

Our findings were consistent with existing evidence in Thailand which demonstrates secular changes in sexual behavior and that the changes are particularly more pronounced in women. In Thailand, the National HIV-related Behavior Sentinel Surveillance has been conducted annually since 1995 by the Bureau of Epidemiology, Ministry of Public Health, among various subpopulations such as military recruits, women attending antenatal care clinics, men attending STIs clinics, etc. and later expanded to include high school and vocational school students in 1996. It has been longitudinally demonstrated that the sexual experience rate of high school students (Grade 11, median age 16–17 years old) is on a continuous rise over the past decade: from 9.8 % in 1996 to 28.0 % in 2011 in men and from 3.5 to 16.4 % in women (Bureau of Epidemiology, 2011).

The changing patterns of sexual behavior in younger generations in our study were consistent with previous population-based sex surveys from industrialized countries conducted in the 1990s. Surveys from Australia (Boyle, Dunne, Purdie, Najman, & Cook, 2003), Britain (Johnson et al., 1994), France (ACSF Investigators, 1992), Japan (Ono-Kihara, 2011), New Zealand (Davis & Lay‐Yee, 1999), Norway (Sundet, Magnus, Kvalem, Samuelsen, & Bakketeig, 1992), Sweden (Giesecke, Scalia-Tomba, Göthberg, & Tüll, 1992), and the United States (Laumann, Gagnon, Michael, & Michaels, 1994; Turner et al., 1995) have confirmed progressive declines in age at first sexual intercourse together with a narrowing gap of gender differences between men and women.

To our knowledge, our study was the first population-based survey to document such changes in Thailand. Such changes in sexual behavior and attitudes are a major public health concern where STIs and unintended pregnancies have been rapidly increasing among adolescents over the past 15 years (Bureau of Epidemiology, 2013; Ministry of Social Development and Human Security, 2010), where there is still an endemic of HIV/AIDS in various subgroups (Armed Forces Research Institute of Medical Sciences, 2011; Bureau of Epidemiology, 2012; UNAIDS, 2012) and where a new wave of HIV is predicted to emerge through 2025 via both heterosexual and homosexual transmission (Commission on AIDS in Asia, 2008). It is well established that younger age of sexual onset is a risk factor for HIV infection (Gregson et al., 2002; Pettifor, 2004; Sarkar et al., 2006; Wand & Ramjee, 2012), other STIs (Celentano et al., 2008; Duncan et al., 1990; Gindi, Erbelding, & Page, 2010; Kaestle, Halpern, Miller, & Ford, 2005), and unintended pregnancy (Ma et al., 2009; Wellings et al., 2001). Furthermore, multiple sexual partnerships are an important determinant of transmission of HIV/STIs (Koumans et al., 2001; Morris & Kretzschmar, 1997; Potterat et al., 1999; Terrault, 2002; Winer et al., 2003). Many nationwide population-based surveys have also demonstrated that such changes in sexual norms are associated with rising incidence of STIs in the United Kingdom (Wellings et al., 2001), unintended pregnancies in the United States (Hofferth, Kahn, & Baldwin, 1987), and induced abortions among adolescents in Japan (Ono-Kihara, 2011).

Concomitant with the changes, however, our data also revealed that the traditional “double standards” of sexual norms is still evident in all age groups: men initiate sexual activity earlier, have more lenient attitudes towards the sex trade, have multiple sexual partnerships, and more commonly engage in casual and commercial sex than women. With regards to attitudes, the overall rate of acceptance of premarital sex in male adolescents was higher than premarital sex in female adolescents and similarly, a higher tolerance of premarital sex in middle-aged men than in middle-aged women. Such double standards in sexual norms in Thailand may also have significant public health implications. The community-wide attitudes toward the sexual activities of young unmarried women may lead them to feel stigmatized and discouraged to seek contraceptives, sexual and reproductive health information, and services (Tangmunkongvorakul et al., 2005; Techasrivichien, 2013) and hence place them at increased risk for adverse sexual health outcomes.

It is true that by virtue of Thailand’s “success” in controlling the HIV outbreak in the 1990s among high risk groups (Ainsworth, Beyrer, & Soucat, 2003; Low-Beer & Sarkar, 2010; Rojanapithayakorn & Hanenberg, 1996), sexually active men and women in Thailand today would likely be by far less at risk than they were several decades ago. Nevertheless, it has been indicated that the “success” in the control of HIV infection through commercial sex does not have much impact on the slow but steady transmission from infected male clients of CSWs to their regular sex partners and the transmission through casual sexual relationships (UNAIDS, 2009; World Health Organization, 2004). The ineffectiveness of existing programs are likely evident by the rising STIs and unintended pregnancies among adolescents since the beginning of the century (Bureau of Epidemiology, 2013; Ministry of Social Development and Human Security, 2010). This is the background of the projections Commission on AIDS in Asia (2008) that, by the year 2025, Asia will face an unprecedented wave of HIV epidemic through sexual transmission. Being among the countries with the highest HIV prevalence in Asia (UNAIDS, 2013), revitalization of existing prevention programs and development of culturally appropriate interventions to prevent adverse sexual health outcomes is, thus, of vital importance and urgently needed in Thailand.

Our research may have implications for other Asian countries undergoing a similar process of urbanization and globalization and that share similar cultural backgrounds and values. Considering emerging attention on premarital sex of young people in many Asian societies (Adhikari & Tamang, 2009; Gipson, Gultiano, Avila, & Hindin, 2012; Jaya & Hindin, 2009; Le Linh, 2009; Tang et al., 2012; Wong, 2012), it is likely that the sexual norms of young people, particularly of young women, is now rapidly changing in many other Asian countries as well. As the process of urbanization is still continuing in Thailand (National Statistical Office, 2011), our study could serve as a baseline to monitor further changes of sexual norms over time. With the aging of the cohorts of our study and the emerging of new young cohorts, it is likely that Thai sexual norms may be virtually transformed in the future.

### Strengths and Limitations

This study was designed to maximize methodological validity. Sampling was by means of multistage probability sampling at a provincial scale with extensive mapping and efforts were made to visit multiple times if participants were not at home. The survey was conducted using a self-administered questionnaire through an internet-enabled tablet to minimize interviewer bias and socially desirable answers on the sensitive issue of sexual behavior. These efforts yielded high overall response rates of 85.5 %. In spite of these efforts, however, bias could have been introduced if nonresponse (15 %) occurred in a nonrandom fashion, being biased to sexually active or inactive subpopulation. Generalization of the results of this study should be done with caution since this study was conducted only in one province of Thailand. Finally, recall biases, especially on the cumulative number of lifetime partners, particularly among older generations, should also be noted.

### Conclusion

We found strong evidence for a decline in reported age of sexual initiation, a higher number of sexual partners, a shift in the type of the first sexual partner, and a greater rate of acceptance of adolescent premarital sex among younger generations. The study highlights profound changes among young Thai women. In contrast to the significant gender gap in older generations, sexual profiles of young Thai women have evolved to resemble those of young men with attitudes gradually converging to similar sexual standards. Our study underscores gender- and generation-differences in sexual norms, which in part, may explain the recent transformations of Thai sexual norms. While also taking into consideration the persistence of a sexual “double standard” between men and women, it is vital to continue monitoring such changes, in light of the potential impact they may have on the course of the HIV/STIs epidemic and unintended pregnancies.
